# Looking ahead in the COVID-19 pandemic: emerging lessons learned for sexual and reproductive health services in low- and middle-income countries

**DOI:** 10.1186/s12978-021-01307-4

**Published:** 2021-12-14

**Authors:** Aduragbemi Banke-Thomas, Sanni Yaya

**Affiliations:** 1 School of Human Sciences, University of Greenwich, London, UK; 2 LSE Health, London School of Economics and Political Science, London, UK; 3 Present Address: School of International Development and Global Studies, Faculty of Social Sciences, University of Ottawa, Ottawa, Canada; 4 The George Institute for Global Health, Imperial College London, London, UK

**Keywords:** Sexual and reproductive health, Family planning, Contraception, Abortion, Gender-based violence, COVID 19, Pandemic, Lessons

## Abstract

The COVID-19 pandemic has caused widespread disruption to essential health service provision globally, including in low- and middle-income countries (LMICs). Recognising the criticality of sexual and reproductive health (SRH) services, we review the actual reported impact of the COVID-19 pandemic on SRH service provision and evidence of adaptations that have been implemented to date. Across LMICs, the available data suggests that there was a reduction in access to SRH services, including family planning (FP) counselling and contraception access, and safe abortion during the early phase of the pandemic, especially when movement restrictions were in place. However, services were quickly restored, or alternatives to service provision (adaptations) were explored in many LMICs. Cases of gender-based violence (GBV) increased, with one in two women reporting that they have or know a woman who has experienced violence since the beginning of the pandemic. As per available evidence, many adaptations that have been implemented to date have been digitised, focused on getting SRH services closer to women. Through the pandemic, several LMIC governments have provided guidelines to support SRH service delivery. In addition, non-governmental organisations working in SRH programming have played significant roles in ensuring SRH services have been sustained by implementing several interventions at different levels of scale and to varying success. Most adaptations have focused on FP, with limited attention placed on GBV. Many adaptations have been implemented based on guidance and best practices and, in many cases, leveraged evidence-based interventions. However, some adaptations appear to have simply been the sensible thing to do. Where evaluations have been carried out, many have highlighted increased outputs and efficiency following the implementation of various adaptations. However, there is limited published evidence on their effectiveness, cost, value for money, acceptability, feasibility, and sustainability. In addition, the pandemic has been viewed as a homogenous event without recognising its troughs and waves or disentangling effects of response measures such as lockdowns from the pandemic itself. As the pandemic continues, neglected SRH services like those targeting GBV need to be urgently scaled up, and those being implemented with any adaptations should be rigorously tested.

## Background

Since the declaration by the World Health Organization (WHO) on 11 March 2020 that the Coronavirus Disease 2019 (COVID-19) outbreak that started in Wuhan, China had gained pandemic status [1], several countries have gone through waves and troughs. Through these times, new variants have emerged while vaccination efforts continue to be ramped up to varying levels of success across and within countries [2]. To date, there have been 265 million confirmed cases and almost 5.4 million deaths because of COVID-19, with about three-fifths of the cases and deaths occurring in low- and middle-income countries (LMICs) [3, 4]. In this time, COVID-19 has caused widespread disruption to essential health services, with a devastating impact on health systems across the globe.

Indeed, concerns regarding the impact that the pandemic could have on sexual and reproductive health (SRH) services, including family planning (FP) and safe abortions, were rife from the beginning of the pandemic [5, 6]. These concerns were further heightened by the restrictions to movement implemented in many countries as part of efforts to limit the spread of COVID-19 [7]. Early in the pandemic, Riley et al. predicted that a 10% decline in the use of contraceptives would result in 49 million additional women with an unmet need for modern contraceptives and 15 million additional unintended pregnancies across 132 LMICs [8]. The authors also predicted that a 10% shift in abortions from safe to unsafe would lead to over three million additional unsafe abortions being performed [8]. For FP, a lot of the concern stemmed from an anticipated disruptive effect of the COVID-19 pandemic on the manufacture and supply of FP commodities, diversion of health care providers, equipment, and facilities to serve COVID-19 cases, service closures and fear of clients about contracting COVID-19 from health facilities [9, 10]. Indeed, these concerns were not have been unwarranted. In the recent past, the 2016 West African Ebola Virus Disease epidemic significantly affected FP services in Liberia and Sierra Leone [11]. In addition, there were concerns amongst researchers and practitioners in the field that the restrictions to movement could lead to increased incidences of gender-based violence (GBV) [5, 12]. There were also concerns raised at the time that GBV services may not be available, and with lockdown, women will be put in a situation in which they will be unable to seek support, access services, and leave their abusers [13]. Others highlighted concerns regarding the possibility that available resources to support victims include hotlines, crisis centres, and counselling services, maybe scaled down because of the pandemic, which will further limit services available to women [14].

It is now over 2 years since the outbreak started. With public health measures such as vaccination being very vital in exiting the pandemic, scientists have feared that if the status quo of vaccination is maintained, COVID-19 might remain endemic in many parts of the world, with LMICs most likely to be the worst sufferers being that vaccination rates in many of these countries remain paltry [2, 15]. Indeed, the emergence of new variants like the Omicron in South Africa, which was recently labelled as a variant of concern by the WHO [16], suggests that any service disruptions that were experienced during the early parts of the pandemic might resurface or remain a feature of many LMICs for a while. As such, it is essential to curate any lessons learnt so far in the provision of essential services, more so SRH services, which remain critical for women during this crisis period. Emerging and re-emerging infectious diseases highlight the potential of aggravation in health inequalities confronted by vulnerable segments of the population, especially women [17]. In this commentary, we take a step back to review the available evidence on the actual impact of the COVID-19 pandemic on SRH service provision and evidence of adaptations that have been implemented to date. These critical *‘live’* lessons can guide SRH service planning in the immediate and distant future.

## Evidenced impact of the COVID-19 pandemic on SRH services in sub-Saharan Africa

Considering the concerns and building on lessons on previous outbreaks, the WHO declared that SRH services were vital and essential services to be maintained during the COVID-19 pandemic [18]. Despite this global declaration, many of the concerns researchers had earlier on have since been realised during this pandemic. Indeed, during the early phase of the pandemic, SRH services were shut down or minimised in many LMIC settings [19]. As per a global survey, 86%, 62% and 46% of a group of clinicians, researchers, and staff of non-governmental organisations (NGOs) from 29 countries, including 12 LMICs, reported that access to FP, surgical and medical abortion services, respectively were reduced or much reduced due to COVID-19 [20]. However, emerging evidence shows that the scale of disruption was not as deleterious as was initially envisaged or at least service provision quickly rebounded to pre-pandemic levels after restrictions to movement were lifted. For example, early in the pandemic, 68% of countries in a global WHO-led survey reported disruptions in FP services [21]; however, services were quickly restored, or alternatives to service provision were explored [22]. The lockdown period in Mozambique was associated with a modest drop in service provision, which was quickly followed by a relatively rapid rebound [22]. The same phenomenon occurred in Nigeria, where frontline health workers also reported a quick rebound in service provision instigated by rapid deployment of various multilevel and health systems responses [23]. As per available evidence, in Nigeria, there was only a slight decrease in the proportion of primary health centres offering FP services from 98% before the pandemic, 95% during the lockdown, to 92% after the lockdown. Though the number of clients who received care halved during the lockdown compared to before the lockdown, there was a 3% increase in cases after lockdown compared to pre-pandemic times [24]. Similarly, in Ethiopia, there was a short-term reduction in adolescent modern contraceptive service provision between March and April 2020 across the public sector and in the country's two largest private sector providers of SRH (3.5% reduction across both public and private sectors) [25].

Analysis of the Performance Monitoring for Action data found non-significant increases in the proportion of women needing contraception in Burkina Faso and Kenya and Kinshasa, Democratic Republic of Congo, with Lagos, Nigeria the only setting showed significantly increased need [26]. As per the same study, there was a 17.4% increase (30.7–48.1%) and 7.4% increase (71.6–78.9%) in the proportion of women of reproductive age who used contraception in rural Burkina Faso and Kenya respectively. Apart from urban Kenya, no significant differences in contraception use were reported in the urban areas studied [26]. A separate study largely agreed that there was no significant difference in contraceptive use before and during the pandemic in Lagos. However, it identified that women in their twenties were 50% less likely to use modern contraceptives during the pandemic than those in their thirties. It also showed that married and divorced women were about three and more than three times more likely to use modern contraceptives during the pandemic compared to single women [27]. In Burkina Faso and Kenya, 69% and 82% of women did not change their contraceptive status during the COVID-19 pandemic and of those who did, more of them adopted a method that was either as or more effective than their pre-pandemic contraception (25% in Burkina Faso and 13% in Kenya) than to discontinue (6.0% and 5.3%, respectively) [28].

Linked to contraception, a study conducted in a community-based pre-exposure prophylaxis programme amongst adolescent girls and young women in South Africa showed that sexually transmitted infection (STI) test positivity increased from 23 to 30% (*p* = 0.20) for Chlamydia trachomatis, 7 to 14% for Neisseria gonorrhoeae (*p* = 0.08), and 8 to 12% for Trichomonas vaginalis (*p* = 0.32). In addition, test positivity for pregnancy increased from 1.2% before to 4.1% during the COVID-19 epidemic (*p* = 0.002) [29]. There were stories of women in Nigeria who could not access contraceptive injections during the lockdown, got pregnant, resulted in unsafe abortion, and almost lost their lives [30].

Regarding safe abortion and post-abortion care, many static clinics in LMICs had to be shut due to strict lockdowns during the early parts of the pandemic [31]. In Ethiopia’s second-largest tertiary and referral hospital, safe abortion services and comprehensive abortion care were reduced by 16.4% and 20.3%, respectively [32]. A report in Ethiopia showed that there was a 9.2% increase in the use of safe abortion care between the onset of the COVID-19 pandemic and the end of 2020. The authors attributed this increment either to an increase in unintended pregnancy, the result of the concerted efforts of the Ministry of Health to increase access to safe abortion care in the country or some combination of both [25]. In Nepal, the number of women who visited one of its largest teaching hospitals for safe abortion services during lockdown was 47.1% lower than after movement restrictions were eased in the country. In the same study, the researchers reported that women presented at a later gestation period during the lockdown with a mean of 9.5 weeks compared to 7.5 weeks following lockdown [33]. According to a Marie Stopes International (MSI) commissioned survey, awareness of available abortions services decreased from 61 to 44% in India before and during the pandemic. A third of women (30%) seeking an abortion reported that their local clinic was closed [34].

For GBV, the United Nations Women in a study conducted in 13 LMICs reported that one in four women say that household conflicts have become more frequent since COVID-19, with one in two women reporting that they have or know a woman who has experienced violence since the COVID-19 pandemic [35]. In countries like Kenya and Morocco, as many as 80% and 69% of women respectively reported that they or a woman they know experienced a form of violence since COVID-19 [35]. In Kenya, 52% and 65% of women who had or knew other women who had experienced a form of violence reported physical and verbal abuse, respectively [36]. In India, the National Commission for Women (NCW), which receives complaints relating to violence against women, recorded a more than two-fold increase in GBV per week comparing weeks before and during the lockdown. Complaints of rape or attempted rape increased steeply from two to 13 over the same period [37]. Jagori, a Delhi-based NGO, which manages helplines for GBV victims, saw a 50% drop in calls, with suggestions that this drop may relate to the lack of privacy that women had from their abusers or their families to be able to place a call [37].

## Adaptations and innovations in SRH services implemented during the COVID-19 pandemic in LMICs

Several adaptations and innovations in SRH service provision have been implemented in LMICs during the pandemic. For example, during the lockdown, MSI approached the government in Madagascar for permission to operate and allow MSI buses to travel on roads to deliver services to women in need at home and transport women to health facilities for free [38]. In addition, in Uganda, MSI worked in partnership with United Nations Population Fund Agency to deliver FP commodities via motorbikes to women who pre-ordered them using a mobile application. MSI adapted practice to set up home-based call centres to provide free SRH advice and service referrals over the phone, WhatsApp, and various social media platforms [38]. These contact centres also reassured clients regarding COVID-19 preparedness and safety of health facilities, thereby helping clients overcome the fear that they might contract COVID-19 if they visited health facilities [31]. During the first month of lockdown, calls to contact centres from clients requesting information increased over three-folds in Ghana [38]. Mobile phones and WhatsApp were also used to provide remote supportive supervision and, in some cases, capacity building to service providers, where these would have been delivered in person before the pandemic [31]. In Nepal, MSI worked with ambulance services and other NGOs to transport medical abortion commodities to local pharmacies [31].

In May 2020, Population Services International (PSI)’s Adolescents 360 (A360) project implemented by Society Family Health launched a Facebook promotion campaign for young women to access reliable on-demand FP information safely. The platform now has over 70,000 followers, has broadened its client base, and allowed more young people to access the digital FP curriculum being offered [39]. Other adaptations offered in Nigeria include the delivery of FP awareness and referrals via WhatsApp [39]. In Kenya, PSI also used WhatsApp and short message services to create awareness regarding the availability of FP services during COVID-19. In addition, community health workers (CHWs) distributed FP products and provided services to rural women who had limited access. This approach led to a 2.5-fold increase in the number of women reached from August to September 2020 [39]. In Uganda, PSI used popular social media platforms like Facebook and Twitter to ensure demand creation for family planning services. In India, PSI partnered with Docterz.com to deliver a bespoke FP counselling and telemedicine service that focused on oral contraception and emergency contraception methods and linked women to an e-pharmacy to purchase their desired product, then subsequently delivered to them at home. As of May 2021, 549 e-consultations with young women have been completed [39].

MSI established telemedicine hubs in Cape Town and Sandton, South Africa, which supported women with self-managed abortion services. Over four months, 1233 women were serviced, with over 1600% increase in clientele per month comparing launch month (April 2020) to July 2020. It was deemed to have a wider reach, less expensive since no travel costs were involved, efficient and client-centred since there was no queuing involved, and it minimised COVID-19 transmission. It also assured the confidentiality of clients [34]. A similar platform was used in Benin to support the delivery of safe abortion and post-abortion care, while in Mozambique, telemedicine was used for follow-up support to confirm the completion of abortion [40]. Though, in some settings, providers reported challenges with access to stable Internet connection [40].

The NCW in India launched a helpline number to enable those experiencing GBV to send a WhatsApp message to access help [41]. There was also recognition of the role of the media in preventing GBV during COVID-19, including protecting victims, highlighting available support services, expressly stating consequences for GBV perpetrators, and monitoring the appropriateness of television content that depicts GBV [42]. The Government of Kenya included telephone numbers to call or SMS that were managed directly by the government and toll-free numbers managed with the support of private partners in its guidelines. The government advised counties that these numbers needed to be published and widely disseminated [43]. Special consideration for the use of emergency contraceptive pills to be promptly given to survivors of sexual and gender-based violence as part of standard post-exposure prophylaxis was also issued by the government [43]. Compared to countries with mildly restrictive abortion laws, countries with severely restrictive abortion laws did not change policy in response to the COVID-19 pandemic [20].

## Reflecting on the adaptations and innovations in SRH services implemented to date

A previous review proposed several interventions that may work for FP provision in the context of the COVID-19 pandemic, including integration of trained CHWs in delivery of a range of FP services depending on national policy, proactively offering immediate postpartum and postabortion FP counselling and services, mobile outreach services or patent and proprietary medicine vendors and pharmacies to deliver FP commodities, leveraging digital health platforms including telemedicine for various aspects of FP service provision from procurement to actual provider–client interphase [44]. At the start of this pandemic, several LMIC governments provided guidelines to support SRH service delivery. For example, the Kenyan government advised facilities to increase minimum stock levels, prescribe less skill-intensive methods such as condoms, pills, and patches, which could be delivered with minimal client-provider interaction. And advised use of telemedicine, where feasible [43]. NGOs working in SRH programming have also played significant roles in ensuring SRH services have been sustained by implementing several adaptations at different levels of scale and to varying success.

Many of these adaptations were implemented based on guidance and best practices and, in many cases, leveraged evidence-based interventions. Indeed, several guidelines and best practices for SRH provision during the pandemic were recommended by health authorities [45]. For example, early in the pandemic, the International Federation of Gynaecology and Obstetrics (FIGO)’s Contraception and Family Planning Committee joined other health authorities to make a case for long-acting reversible contraceptives, highlighting their value in guaranteeing user independence, minimising the need for repeated re-supplies and ability to be safely provided with the use of adequate personal protective equipment (PPE) [46]. Based on the evidence, some NGOs have provided access to Misoprostol. Even before the pandemic, there was clear evidence that there is no difference in the safety, effectiveness, and acceptability of home-based medical abortion with Misoprostol compared to clinic-based medical abortion in LMICs [47]. Indeed, there is significant evidence that Misoprostol, when used appropriately, will lead to the successful termination of 80–95% of pregnancies without requiring further surgical intervention [48]. There is also evidence that shows that in LMICs, unsafe abortion could be as much as 20 times more expensive for patients compared to safe abortion [49].

Some interventions were also the sensible thing to do. For example, though FP utilisation did not massively reduce, especially in urban areas, there was still some reduction [26], and lockdown at home limited use for younger women who were living at home with their parents [27]. In addition to other factors, the fear of contracting the virus while engaging with health facilities was reported by frontline health workers across the globe [50] and in local surveys of women themselves. As many as three in four women expressed this fear in a study conducted in Lagos, Nigeria [27]. With a potential fourth wave on the way and uncertainty on the potency/severity of future variants, attention needs to be firmly placed on getting care to women, especially if they do not need to come to the care itself.

Others, though sensible, need to be contextualised. It can indeed be argued that, especially during these challenging times for humanity, home-based self-abortion care will afford health systems the respite needed in having to manage patients who would otherwise not have been required to visit health facilities. For the patients themselves, it minimises the cost of accessing the care that they need. This proposition was well laid out in a “love letter” to Misoprostol early in the pandemic [51]. However, restrictive abortion laws, political will, regulatory challenges, weak infrastructure, and finances limit effectiveness that may be achieved from using telemedicine to support safe abortion during the COVID-19 pandemic [52]. It is encouraging to note that MSI is exploring the possibility of implementing home-based self-abortion in their programmes in Ethiopia, Ghana, Mexico, and South Africa. The reality of this remains to be seen, with policy restrictions in some countries banning self-administration altogether [31]. Initial pilot successes in South Africa offer some hope [34].

While concerns about a rise in GBV have been matched by an evidenced increase in GBV evidenced across many countries, interventions preventing GBV or supporting victims have not been widely reported in the literature. More concerning is that these have not been tested for effectiveness. It is certainly not enough to create a platform without showing if it works. Indeed, recommendations that medical, legal, and policy mechanisms for GBV victims must be retained during the pandemic were made by several agencies of the United Nations, colleges, faculties and societies of obstetrics and gynaecology across the globe [45]. In addition, NGOs and other advocates have pushed governments to act, including proposing recommendations on what needs to be done [53]. In reviews published before the pandemic, only a handful of interventions to address GBV have been tested in LMICs [54, 55]. More importantly, none of these interventions included the use of helplines, social media or digital platforms that have become popular during the pandemic.

Across the board, many actions targeted at SRH service provision have been documented by the private sector with limited evidence on adaptations and innovations within the public sector, which has reported several adaptions for maternal and newborn health [56, 57]. For example, many self-care methods were driven by the private sector. While this is not surprising, it might also lead to equity issues [58]. Furthermore, a lot of the focus on evaluating these adaptations and innovations has been on service outputs. However, what remains unclear in the existing literature is how well the adaptations and innovations implemented have worked in achieving outcomes of service delivery (effectiveness). In addition, critical gaps remain regarding implementation outcomes such as cost, acceptability, feasibility, and sustainability [59]—all of which are crucial considerations for delivering successful SRH services. Even for Misoprostol, where there is evidence of acceptability [47], this evidence pre-dates the pandemic, which is a hugely different scenario. The pandemic, combined with a lockdown and limited access to care, women might make other choices regarding acceptability. This possibility underscores the urgent need for interventions to be tested within the context of the ongoing crisis.

## Critical actions needed for SRH policy, programming, and research as the pandemic continues

Hopefully, LMIC health systems have learned from the challenges in accessing PPEs during the first year of the pandemic, when some hospitals required philanthropic donor support to procure PPEs for providers [60]. Going forward, the supply chain for SRH commodities in LMICs needs to be sustained through this pandemic to ensure no reversal in the stability achieved to date. PPE must be continuously enforced for provider–client engagements, and reassurance of communities will be crucial. In terms of service delivery, integration of CHWs in SRH service provision worked well in realising service outputs in Kenya [39]. However, isolated investments in CHWs for COVID-19 will not be enough [61, 62]. SRH services provided in health facilities remain important and those women who did were still satisfied with the care received [63]. The public sector can do more regarding access to SRH commodities. SRH targeted vouchers and subsidies provide a vehicle to support women during this crisis period [58], especially if lockdowns that have a massive impact on the economic livelihood of families are instituted. More needs to be done to support GBV. Social media has played an essential role in saving victims in high-income settings, with simple hand signals [64] that can certainly be promoted in LMICs. As all the various actions are implemented, efforts need to be made to map them during this pandemic, as is being done for safe abortion with the COVID-19 safe abortion response map.

As in the Ebola outbreak, during this COVID-19 pandemic, it seems that though there were service disruptions, “to a large extent, it was not health service provision that failed” [65]. However, the Ebola outbreak did not involve multiple waves of disease like is evident in the COVID-19 pandemic. In addition, access to SRH commodities for vulnerable subpopulations like young people has been affected differently than older women, especially when additional constraints like lockdowns are in place [27]. As such, any implementation approaches must segment populations and meet their specific needs as we move forward. Platforms such as the Modelling the Impact of COVID-19 on Reproductive Health Outcomes (MICRO)’s Model of the Impact of COVID-19 Mitigation on contraceptive needs can serve as an excellent foundation to build on for scenario planning and quantification [58], as countries implement strategies to meet contraceptive needs of populations.

In terms of research, many of the gaps in knowledge relating to the impact of COVID-19 on SRH set at the beginning of the pandemic [66] have now been answered. Indeed, we know the impact COVID-10 has had on SRH services. However, as the pandemic progresses, it is vital to recognise the varying patterns of this outbreak and tailor SRH service provision and research accordingly. The COVID-19 pandemic has not been a homogenous experience for all LMICs, and even within the same country, it has not been the same experience over time. For example, the impact of lockdown on FP services may not have been as hard on clients in Mozambique, where restrictions were not as stringent [22]. Where possible, it is essential to disentangle the impact of the pandemic on SRH service provision from that of the lockdown.

Another critical gap in knowledge that is yet to be addressed relates to robust evidence on how well the adaptations and innovations have worked. Generally, the adaptations implemented thus far have been mostly digitised and focused on getting SRH services closer to women. Where digital platforms have been implemented, some have also served the pandemic itself, providing additional benefits than intended. These adaptations must be tested using robust methods, including quasi-experimental studies and step-wedge designs. These should be supported with implementation research methods to help us understand key implementation outcomes and substrates for successful implementation and value for money assessments [67, 68]. Such evidence will be essential in guiding future SRH service provision. The pandemic offers us a once in a lifetime opportunity to test interventions, adaptations, and approaches that can work in such a crisis at a large scale and over a long time, with the pandemic not abating after 2 years.

## Abbreviations

COVID-19: Coronavirus disease 2019
FP: Family planning
LMICs: Low- and middle-income countries
GBV: Gender-based violence
MSI: Marie Stopes International
NCW: National Commission for Women
PPE: Personal protective equipment
PSI: Population Services International
SRH: Sexual and reproductive health
WHO: World Health Organization
